# Can Hepatitis C Virus (HCV) Direct-Acting Antiviral Treatment as Prevention Reverse the HCV Epidemic Among Men Who Have Sex With Men in the United Kingdom? Epidemiological and Modeling Insights

**DOI:** 10.1093/cid/ciw075

**Published:** 2016-02-16

**Authors:** Natasha K. Martin, Alicia Thornton, Matthew Hickman, Caroline Sabin, Mark Nelson, Graham S. Cooke, Thomas C. S. Martin, Valerie Delpech, Murad Ruf, Huw Price, Yusef Azad, Emma C. Thomson, Peter Vickerman

**Affiliations:** 1 Division of Global Public Health, University of California San Diego; 2 School of Social and Community Medicine, University of Bristol; 3 University College London; 4 Chelsea and Westminster Hospital; 5 Imperial College; 6 Public Health England, Colindale; 7 Medical Affairs, Gilead SciencesLtd, London; 8 Mid Essex Hospital Services NHS Trust, Chelmsford; 9 National AIDS Trust, London; 10 MRC University of Glasgow Centre for Virus Research, United Kingdom

**Keywords:** hepatitis C virus, HIV, men who have sex with men, antiviral treatment, prevention

## Abstract

***Background.*** We report on the hepatitis C virus (HCV) epidemic among human immunodeficiency virus (HIV)-positive men who have sex with men (MSM) in the United Kingdom and model its trajectory with or without scaled-up HCV direct-acting antivirals (DAAs).

***Methods.*** A dynamic HCV transmission model among HIV–diagnosed MSM in the United Kingdom was calibrated to HCV prevalence (antibody [Ab] or RNA positive), incidence, and treatment from 2004 to 2011 among HIV-diagnosed MSM in the UK Collaborative HIV Cohort (UK CHIC). The epidemic was projected with current or scaled-up HCV treatment, with or without a 20% behavioral risk reduction.

***Results.*** HCV prevalence among HIV-positive MSM in UK CHIC increased from 7.3% in 2004 to 9.9% in 2011, whereas primary incidence was flat (1.02–1.38 per 100 person-years). Over the next decade, modeling suggests 94% of infections are attributable to high-risk individuals, comprising 7% of the population. Without treatment, HCV chronic prevalence could have been 38% higher in 2015 (11.9% vs 8.6%). With current treatment and sustained virological response rates (status quo), chronic prevalence is likely to increase to 11% by 2025, but stabilize with DAA introduction in 2015. With DAA scale-up to 80% within 1 year of diagnosis (regardless of disease stage), and 20% per year thereafter, chronic prevalence could decline by 71% (to 3.2%) compared to status quo in 2025. With additional behavioral interventions, chronic prevalence could decline further to <2.5% by 2025.

***Conclusions.*** Epidemiological data and modeling suggest a continuing HCV epidemic among HIV-diagnosed MSM in the United Kingdom driven by high-risk individuals, despite high treatment rates. Substantial reductions in HCV transmission could be achieved through scale-up of DAAs and moderately effective behavioral interventions.

## Abstract (precis)

Epidemiological data and modeling suggest a continuing hepatitis C virus (HCV) epidemic among human immunodeficiency virus-diagnosed men who have sex with men in the United Kingdom. Substantial reductions in HCV transmission could be achieved through scale-up of HCV treatment and behavioral intervention.

An epidemic of hepatitis C virus (HCV) among human immunodeficiency virus (HIV)–positive men who have sex with men (MSM) [1, 2] has been documented in cities in Europe, Australia, and the United States, but with little evidence of transmission among HIV-uninfected MSM [3, 4]. One of the key hubs of this epidemic is London [2]. However, the state and future of the UK epidemic is uncertain, with reported incidence based on case notifications instead of longitudinal cohort trends [5, 6].

Modeling indicates that HCV antiviral treatment for those at risk of transmission such as people who inject drugs could have a primary prevention benefit [7–10]. HIV-positive MSM may be the ideal population to assess HCV treatment as prevention (particularly with interferon [IFN]–free direct-acting antiviral therapies [DAAs], which are highly effective in this population [11, 12]), because most patients are linked to care and frequently HCV tested, and the absolute numbers of HCV/HIV-coinfected MSM are small. However, high reinfection rates (8–15/100 person-years [PY] [13–15]) among HIV-positive MSM might limit the prevention benefits of HCV treatment.

To explore the potential impact of new treatments and other interventions on this epidemic, we took advantage of detailed available UK data and developed a dynamic model of HCV transmission among HIV-positive MSM in the United Kingdom, to assess the epidemic trajectory and project the impact of scaled-up HCV treatment as prevention.

## METHODS

### Epidemiological Data Analysis

The UK Collaborative HIV Cohort (UK CHIC) study is an ongoing observational study collecting clinical data from 16 HIV treatment centers across the United Kingdom [16]. Between September 2012 and September 2013, additional data were collected on HCV treatment from 11 participating centers. Individuals were included in the analysis if they had ever attended one of the 11 centers since 2004, had an HCV antibody (anti-HCV) or RNA test during follow-up, and were recorded as having acquired HIV through sex between men.

Cumulative HCV prevalence was calculated yearly as the number of men who had ever had a positive anti-HCV or HCV RNA test by the end of that year as a proportion of all those who had been tested by that time. Incident infection was assessed among individuals with a negative anti-HCV test and either negative or missing HCV RNA test after 1 January 2004 and at least 1 further test for anti-HCV or HCV RNA. Individuals were followed up until a positive anti-HCV or HCV RNA test or their last date seen at a UK CHIC center. The incidence rate was calculated by dividing the total number of incident infections (any positive anti-HCV or HCV RNA test) by the total number of PY of follow-up. Receipt of HCV treatment (IFN [pegylated or nonpegylated] with or without ribavirin [RBV], telaprevir, or boceprevir) was assessed among all men who had ever received a positive HCV RNA result.

### Mathematical Model

**Figure 1.**
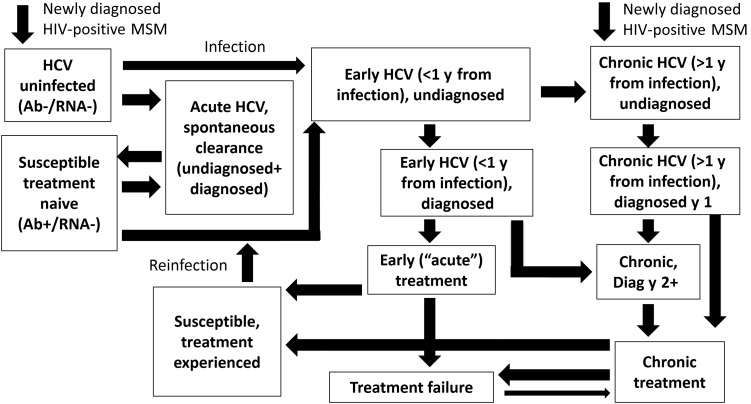
Mathematical model schematic. The model is also stratified by treatment-naive, interferon-experienced, direct-acting antiviral–experienced, and low-/high-risk states. Human immunodeficiency virus (HIV) and non-HIV death occurs from all states. Abbreviations: Ab, antibody; HCV, hepatitis C virus; MSM, men who have sex with men.

We developed a dynamic, deterministic model of HCV transmission, progression, and treatment among diagnosed HIV-positive MSM (Figure 1). Individuals enter at HIV diagnosis, a small proportion with existing HCV coinfection. As the model is dynamic, an individual's risk of acquiring HCV is related to background HCV prevalence and his or her risk behavior. The model tracks HCV disease progression and is stratified by HCV diagnosis status, treatment history, and transmission risk (high/low, based on factors associated with high risk of HCV acquisition among MSM such as injecting drug use and methamphetamine use [17, 18]). We assume MSM who inject do so with other MSM, based on phylogenetic evidence indicating that HCV MSM strains are clustered separately from people who inject drugs [19]. For our baseline analysis, we assume that HCV-uninfected, HIV-diagnosed MSM are only at risk of HCV acquisition from HIV-diagnosed MSM because of the low HCV prevalence among HIV-uninfected MSM and HIV-positive undiagnosed MSM, proportional mixing between risk groups, and movement between high/low risk.

### Model Parameterization and Calibration

The model was calibrated to annual UK CHIC data on HCV incidence, prevalence (Ab or RNA positive) and proportion ever treated among diagnosed HIV-positive MSM in the United Kingdom from 2004 to 2011, and parameterized by data among HIV-diagnosed MSM in the United Kingdom (see list of parameters in Supplementary Table 1). The model was also calibrated to estimate HCV reinfection incidence among HIV-positive MSM in London (7.8/100 PY [95% confidence interval {CI}, 5.8–10.5/100 PY] across 2004–2012) [14] and the size of the HIV-diagnosed MSM population in 2013 [20]. Model projections were validated against annual size estimates of the HIV-diagnosed MSM population from 2001 to 2013 [20, 21].

Based on UK CHIC data, we model treatment rates (from 2003 onward) of 46% (95% CI, 40%–53%) and 22% (95% CI, 20%–24%) treated within 1 year of an acute and chronic diagnosis, respectively. Using these rates and the cumulative proportion ever treated by 2011 (44%), the model estimates an annual treatment rate after the first year of diagnosis of 6.8% (95% CI, 3.8%–9.9%). Sustained virological response (SVR) rates for IFN-based therapy among HIV-positive individuals came from a published meta-analysis [22]; we assume 90% SVR with DAAs. We increased life expectancy from HIV diagnosis over calendar time based on UK data reflecting earlier diagnosis/treatment and more effective antiretroviral therapy [23], and include excess liver-related mortality for MSM coinfected with HCV [24, 25].

To incorporate parameter uncertainty, 1000 parameter sets were randomly sampled from the parameter distributions shown in Supplementary Table 1.

### Intervention Scenarios and Sensitivity Analyses

We model the UK epidemic from 1996 to 2015, assessing the population-attributable fraction of being high risk by assessing the relative difference in cumulative new infections from 2015 to 2025 if the relative risk between high and low risk is set to 1 from 2015 and assuming status quo treatment rates and SVR. We explore the 10-year impact (to 2025) on HCV (Ab or RNA positive) prevalence, chronic (RNA positive) prevalence, primary incidence, and numbers treated for the following scenarios:

- Baseline status quo with IFN/RBV: continuation of current treatment rates and SVR;
- Current treatment rates with DAAs for all: continuation of current treatment rates with DAAs (90% SVR) from 2015;
- DAA scale-up at diagnosis: scale-up DAA treatment rates to 60%/80%/100% treated within 1 year of diagnosis from 2015;
- DAA scale-up to all: scale up DAA treatment rates to 80% treated within 1 year of diagnosis, and 20% per year thereafter from 2015;
- DAA scale-up to all and behavioral intervention: as above and 20% behavioral risk reduction from 2015; and
- No historical treatment from 1996.

**Table 3.** Mathematical Modeling Scenarios

Model Scenario	SVR <1 y From HCV Infection (Sampled Range)	SVR >1 y After Acute Infection (Sampled Range)	Proportion Treated After Acute Diagnosis (Sampled Range)	Proportion Treated the First Year After Chronic Diagnosis (Sampled Range)	Proportion Treated Thereafter	Behavioral Intervention
Baseline status quo with IFN/RBV	80% (70%–90%)	30% (25%–35%)	46% (40%–53%)	22% (20%–24%)	Mean 5.9% (2.5%–97.5% fits 3.5–10)	No
Current treatment with DAA for all	90%	90%	As in baseline	As in baseline	As in baseline	No
DAA scale-up at diagnosis	90%	90%	60%/80%/100%	60%/80%/100%	As in baseline	No
DAA scale-up to all	90%	90%	80%	80%	20%	No
DAA scale-up to all and behavioral intervention	90%	90%	80%	80%	20%	20% reduction in risk for all
No historical treatment	NA	NA	0% (no treatment from 1996)	0% (no treatment from 1996)	0% (no treatment from 1996)	No

Abbreviations: DAA, direct-acting antiviral; HCV, hepatitis C virus; IFN/RBV, pegylated interferon + ribavirin; NA, not applicable; SVR, sustained virological response.

We allow retreatment with DAAs for those who have previously failed IFN-based therapies and those who are reinfected.

One-way sensitivity analyses explore the impact of variations in SVR, retreatment eligibility, HCV testing rates, risk reductions posttreatment (50% and 100%) or postdiagnosis (20% for 1 year or until HCV treatment), assortative mixing, and seeding of HCV from outside the HIV-diagnosed population on the mean chronic HCV prevalence in 2025 for the DAA scale-up to all scenario (Supplementary Data).

## RESULTS

### Epidemiological Data From UK CHIC

Nearly all (98%) of the MSM in UK CHIC under follow-up in 2011 had been tested for HCV (Table 1); the proportion of MSM not known to be infected who were annually HCV tested increased from 31% in 2004 to 65% in 2011 (Supplementary Table 2). The median number of diagnostic tests until the first positive result per individual was 4 (interquartile range, 2–6).

**Table 1.** Cumulative Prevalence (Antibody or RNA Positive) of Hepatitis C Among Human Immunodeficiency Virus (HIV)-Positive Men Who Have Sex With Men in the UK Collaborative HIV Cohort Study

Year	Total No. of MSM Under Follow-up in That Year in UK CHIC	Total No. of MSM Under Follow-up in That Year With a Reported Test by End of Year	% With an HCV Test Reported by End of That Year	Cumulative No. HCV Infected (Ab or RNA Positive)	Cumulative HCV Prevalence (Ab or RNA Positive), %
2004	11 012	6774	61.51	492	7.26
2005	11 765	8398	71.38	641	7.63
2006	12 335	9550	77.42	752	7.87
2007	12 895	10 808	83.82	896	8.29
2008	13 262	11 799	88.97	1049	8.89
2009	13 693	12 607	92.07	1195	9.48
2010	14 147	13 369	94.50	1293	9.67
2011	13 101	12 789	97.62	1261	9.86
Ever	17 574	16 533	94.08	1673	10.12

Abbreviations: Ab, antibody; HCV, hepatitis C virus; MSM, men who have sex with men; UK CHIC, Collaborative HIV Cohort.

The cumulative HCV prevalence (Ab or RNA positive) among HIV-positive MSM increased from 7.26% in 2004 to 9.86% in 2011 (Table 1). A total of 11 386 MSM, who were initially HCV uninfected and who had at least 1 further test during a median of 5 years’ follow-up, were included in the incidence analysis, contributing 54 619 PY of follow-up. Incidence rates from 2004 to 2011 were relatively flat, varying from 1.02 to 1.38/100 PY of follow-up (Table 2).

**Table 2.** Incidence of Hepatitis C Among Human Immunodeficiency Virus (HIV)-Positive Men Who Have Sex With Men in the UK Collaborative HIV Cohort Study

Year	PY of Follow-up of Those HCV Ab Negative	New Infections	Incidence/100 PY of Follow-up (95% CI)
2004	1454	15	1.03 (.58–1.70)
2005	4179	51	1.22 (.91–1.60)
2006	6076	62	1.02 (.78–1.31)
2007	7484	103	1.38 (1.12–1.67)
2008	8752	106	1.21 (.99–1.46)
2009	9405	111	1.18 (.97–1.42)
2010	9782	101	1.03 (.84–1.25)
2011	7487	80	1.07 (.85–1.33)

Abbreviations: Ab, antibody; CI, confidence interval; HCV, hepatitis C virus; PY, person-years.

A total of 1403 MSM had ever received a positive RNA result and were considered eligible for HCV treatment. Of these, 36 individuals were excluded as their treatment dates were prior to their first positive HCV tests. Therefore, a total of 1367 MSM were eligible for inclusion in this analysis. Overall, 586 of 1367 (43%) were ever treated, the majority (60%) of treatments occurring within 1 year of diagnosis (Supplementary Tables 3 and 4).

### Modeling Projections

**Figure 2.**
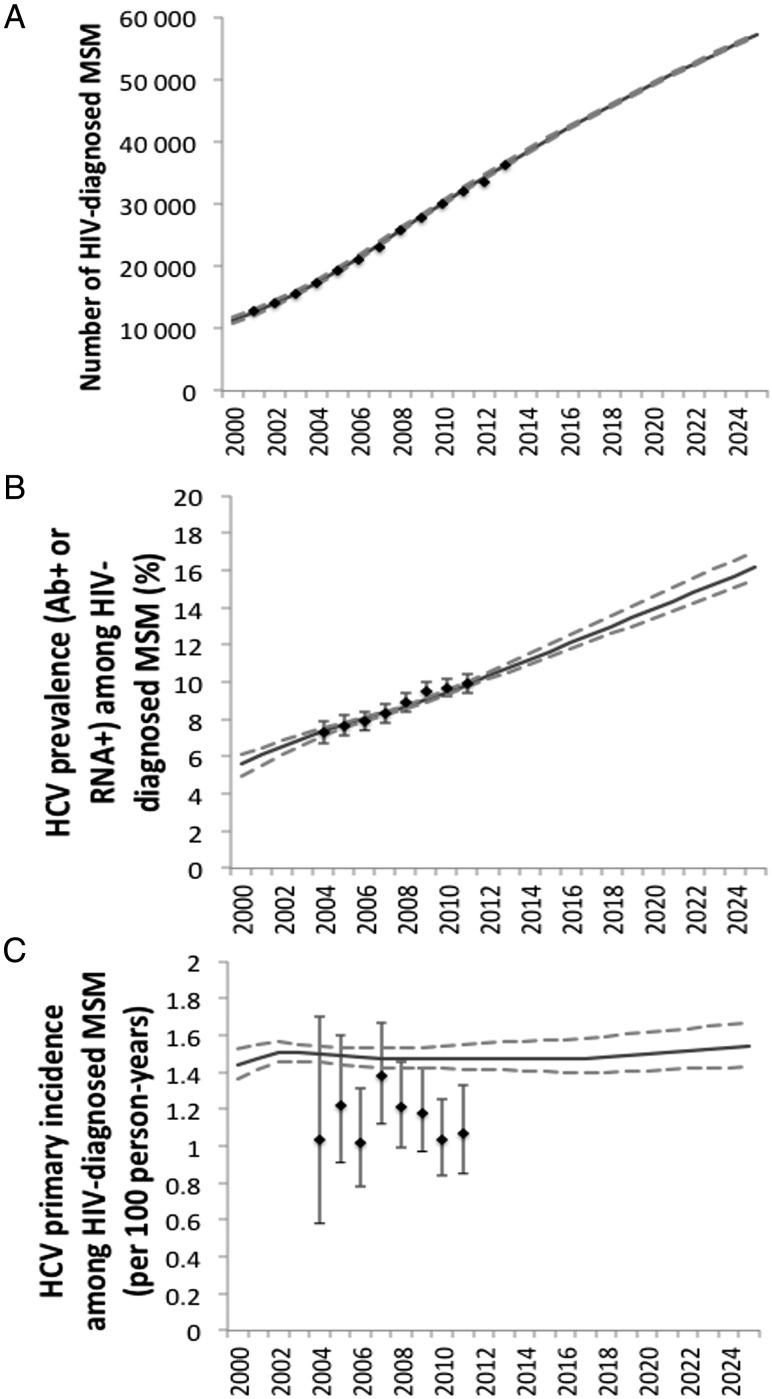
Model fits to epidemiological data from the United Kingdom. *A*, Number of human immunodeficiency virus (HIV)-diagnosed men who have sex with men (MSM). *B*, Hepatitis C virus (HCV) prevalence (antibody [Ab] or RNA positive) among diagnosed HIV-diagnosed MSM. *C*, HCV primary incidence among diagnosed HIV-diagnosed MSM. Solid lines show the mean value of all 1000 simulations; dashed lines show the 2.5% and 97.5% range of the projections. Black diamonds show data from Public Health England (*A*; model calibrated to 2013 value, other values shown for validation) and UK Collaborative HIV Cohort (*B* and *C*; model calibrated against all data points).

The model fits closely matched the number of HIV-diagnosed MSM from 2000 to 2013 (Figure 2*A*) and HCV prevalence (Ab or RNA positive) from 2004 to 2011 (Figure 2*B*). The projected HCV incidence (1.47/100 PY) was toward the upper bounds of the UK CHIC data (Figure 2*C*), and projected reinfection incidence (mean, 7.8/100 PY for 2004–2012) was consistent with UK data [14]. In 2015, the modeled reinfection incidence ranged from 4- to 7-fold that of the primary incidence.

### Population-Attributable Fractions

The model fits estimate a high-risk population size of 7% (2.5%–97.5% interval [95% CI, 3%–14%]), consistent with the estimated proportion of HIV-positive MSM in the United Kingdom reporting injecting drug use or methamphetamine use in the previous 4 weeks [26]. These high-risk individuals contribute more than one-third of prevalent (37% [95% CI, 21%–64%]) and incident (36% [95% CI, 13%–78%]) infections in 2015. Over the next decade, 94% (95% CI, 91%–97%) of infections are attributable to high-risk individuals.

### Projections of Intervention Impact to 2025

A summary of the modelled scenarios can be found in Table 3.

#### Treatment With IFN/RBV

**Figure 3.**
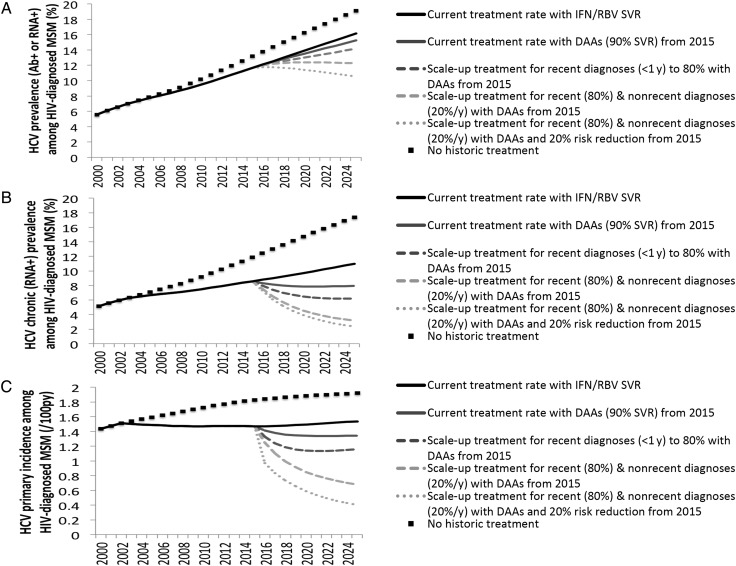
Model projections (mean value of 1000 simulations shown) with various treatment scenarios in the United Kingdom. *A*, Hepatitis C virus (HCV) prevalence (antibody [Ab] or RNA positive) among human immunodeficiency virus (HIV)–diagnosed men who have sex with men (MSM). *B*, HCV chronic (RNA) prevalence among HIV-diagnosed MSM. *C*, HCV primary incidence among HIV-diagnosed MSM. Abbreviations: DAA, direct-acting antiviral; IFN/RBV, interferon/ribavirin; py, person-years; SVR, sustained virological response.

**Figure 4.**
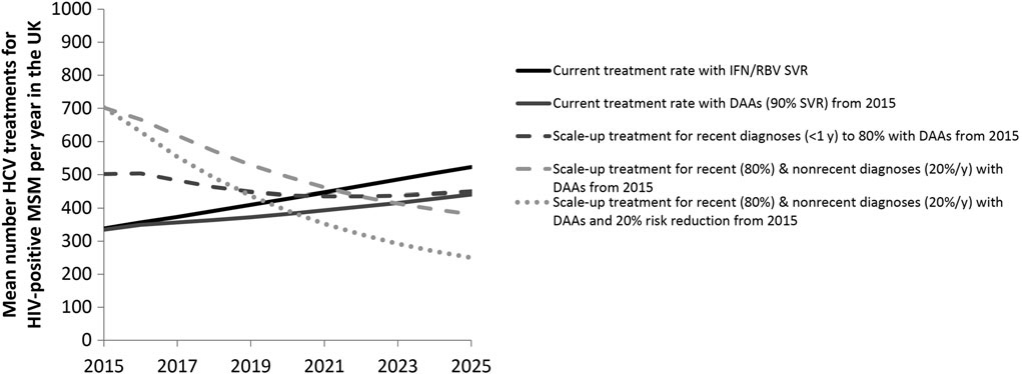
Model projections of the mean number of hepatitis C virus (HCV) treatments for human immunodeficiency virus (HIV)-diagnosed men who have sex with men (MSM) in the United Kingdom for different treatment scenarios. Abbreviations: DAA, direct-acting antiviral; IFN/RBV, interferon/ribavirin; SVR, sustained virological response.

If HCV treatment and SVR rates remain unchanged, the model predicts steadily increasing anti-HCV prevalence, and increasing chronic (RNA positive) prevalence from 8.6% (95% CI, 8.1%–9.1%) in 2015 to 11% (95% CI, 9.9%–12.1%) in 2025 (Figure 3*A* and 3*B*). Due to the expanding epidemic, status quo treatment rates result in greater treatments required yearly (Figure 4). In contrast, incidence will remain relatively flat, at 1.5/100 PY (95% CI, 1.4–1.7/100 PY) in 2025 (Figure 3*C*). However, if there was no treatment, chronic prevalence would have been more than one-third (38%) higher in 2015 (11.9% [95% CI, 11.1%–12.6%]), and 17.4% (95% CI, 15.8%–18.6%) in 2025 (Figure 3*B*). Similarly, incidence would have been 24% higher (1.8/100 PY [95% CI, 1.6–2/100 PY]).

#### Treatment With DAAs

If DAAs are provided from 2015 at current treatment rates, chronic prevalence will remain virtually unchanged over the next decade (8% [95% CI, 7.4%–8.6%] in 2025), but could be a relative 27% lower in 2025 than if IFN/RBV is used (Figure 3*B*). Modest reductions in HCV incidence would be achieved (1.3/100 PY [95% CI, 1.2–1.4/100 PY] in 2025) (Figure 3*C*).

#### Treatment Scale-up With DAAs

Substantial reductions in chronic prevalence can be achieved through scale-up of DAAs (Figure 3*B*). If 60%, 80%, or 100% of recently diagnosed (<1 year) individuals are treated the year of diagnosis (compared with 46% at baseline) but there is no change in treatment rates for nonrecent diagnoses (>1 year), HCV RNA prevalence in 2025 could decrease to 7.4% (95% CI, 6.7%–8.1%), 6.2% (95% CI, 5.6%–7%), or 5.0% (95% CI, 4.4%–6%), respectively (a 33%, 44%, or 55% relative reduction compared with baseline, respectively). Similarly, incidence in 2025 could decline relatively by 15%, 25%, and 36% compared with baseline, respectively. These treatment increases result in 15%, 30%, and 41% greater numbers treated for the first year, respectively, but the annual numbers treated drop below the status quo scenario by 2022 (Figure 4).

More impact is achieved if treatment is scaled up among those with recent (<1 year) and nonrecent (>1 year) diagnoses. If 80% of recent diagnoses and 20% per year of nonrecent diagnoses are treated (compared with 46% and 7%, respectively, at baseline), RNA prevalence could decline to 3.2% (95% CI, 2.8%–4.1%) by 2025 (71% lower than 2025 baseline), and incidence could decline to 0.7/100 PY (95% CI, 0.6–1/100 PY) (56% lower than 2025 baseline). Treatment numbers double the first year, but drop quickly, approaching the status quo scenario by 2022 (Figure 4).

If DAA scale-up (80% <1 year from diagnosis and 20% per year thereafter) is combined with a behavioral intervention that reduces transmission risk by 20% from 2015, HCV incidence decreases by 20% within 1 year to 1.2/100 PY (95% CI, 1.1–1.3/100 PY), and to 0.4/100 PY (95% CI, 0.3–0.7/100 PY) by 2025 (Figure 3*C*). This combined prevention intervention reduces chronic prevalence to 2.4% (95% CI, 2.1%–3.3%) by 2025 (Figure 3*B*) and lowers the annual number of treatments (Figure 4).

### Sensitivity Analysis

Across our sensitivity analyses, all scenarios predict a chronic RNA prevalence of <4% in 2025 with DAA scale-up to all (compared to 3.2% for base case). Less impact (35% relative reduction in chronic prevalence at 2025 compared to base case) is achieved with no retreatment because high treatment rates are not sustainable due to many MSM already being treated. Although greater impact occurs if risk reductions occur posttreatment from 2015 (20% greater impact if risk is reduced by 100%), the effect is limited as retreatment of reinfections is high. Little additional impact (<3% relative difference) is achieved with a short-term (<1 year) 20% reduction in risk behavior after diagnosis; more substantial impact by 2025 occurs with a sustained behavioral intervention targeting all MSM (chronic RNA, 2.4% in 2025) than a short-term intervention targeting those postdiagnosis (chronic RNA, 3.1% in 2025). Little difference (<15% relative difference) is seen with varied SVR, scaled-up diagnosis, partial assortative mixing of high risk, or if HCV infections are seeded into the population (Supplementary Figure 1).

## DISCUSSION

HCV prevalence (Ab or RNA positive) among HIV-diagnosed MSM in the UK CHIC study is projected to increase under current treatment rates from 9.9% in 2011 to 11% by 2025. We estimate that a small high-risk group (<10%) contributes >90% of HCV infections over the next decade. To substantially reduce chronic prevalence (<3%), treatment scale-up among all diagnosed individuals is required, with behavior change interventions necessary to achieve immediate reductions in HCV incidence. The scaled-up rates we examine translate to a maximum of double the numbers of HIV-positive MSM treated (700/year in the United Kingdom) compared to the status quo initially, but these numbers drop below status quo levels by 2022 due to prevention benefits.

To our knowledge, this is the first study to model the HCV epidemic among HIV-positive MSM. Although our analysis is UK-focused, other settings have similar incidence [27–29]. The stable incidence levels found in the United Kingdom are similar to those in Amsterdam [29] and the United States [30], whereas increasing incidence is reported in Switzerland [28]. Given its large size and wide representation of UK clinics, UK CHIC is broadly representative of people living with HIV and attending for HIV care in the United Kingdom. Our UK CHIC estimate is slightly higher than reported previously in the United Kingdom [5, 6], based on case notification data, but also slightly lower than projected by our modeling. Two potential sources of underestimation by UK CHIC data could be due to incident infections without a previous negative test being excluded, or follow-up time being overestimated for patients who cycle in and out of UK CHIC clinics, which, if occurring among higher-risk individuals, could lead to true incidence being underestimated. On the other hand, it is possible those tested are at higher risk of infection, which would overestimate true incidence.

The model projections are limited by several sources of uncertainty that remain even after the uncertainty analyses. First, we model HCV transmission among HIV-diagnosed MSM only, although we include inflow of HIV/HCV-coinfected individuals at HIV diagnosis who are unaffected by our interventions. It is possible that interventions for HIV-diagnosed MSM would also reduce incidence among HIV-undiagnosed MSM, in which case we would expect more impact than shown. Additionally, our sensitivity analysis suggests that seeding of HCV infections from HIV-undiagnosed or HIV-uninfected individuals would have minimal impact. It is unclear whether the higher HCV prevalence among HIV-diagnosed MSM compared with that among HIV-undiagnosed or HIV-uninfected individuals is related to changing risk behavior upon HIV diagnosis, a longer time at risk, or individuals with elevated risk behaviors compared with the general MSM population acquiring both HIV and HCV.

Second, there are limited data defining HCV-related risk behaviors among HIV-positive MSM, and therefore we allowed details of the high-risk population (size, relative risk, time at risk) to vary as part of the model calibration. Additionally, although we include behavioral heterogeneity, we do not explicitly model the transmission network. It is possible that highly connected superspreaders are responsible for many HCV transmission events and should be targeted for prevention. Similarly, we neglect international migration/travel due to a lack of available data, movement that could seed infections and limit the impact of localized interventions. Better epidemiological data on these factors is critical to strengthening the model predictions.

Third, we explore a hypothetical 20% effective behavioral risk intervention, which was not based on a proven intervention in this population. Unfortunately, there is no empirical evidence that this level of HCV risk reduction is achievable. A Cochrane review found evidence for the effectiveness of behavior change interventions (eg, counseling, social and behavioral support) to reduce unprotected anal sex among MSM, reporting an overall reduction by 27% (95% CI, 15%–37%) [31]. These interventions, though primarily aimed at reducing HIV risk, could be effective for HCV as well. Additionally, among people who inject drugs, opiate substitution therapy and high-coverage needle and syringe programs can reduce an individual's risk of HCV acquisition by 50% alone, or 80% in combination [32], but it is unclear how applicable these interventions are to the HCV epidemic among MSM. It is possible that both sexual and injecting-related interventions could play an important role, such as prevention messaging training among sexual health/HIV clinic staff and the distribution of safe “chemsex” kits. One UK clinic is currently examining the impact of club drug behavior change intervention among MSM, but the impact is uncertain at present.

Fourth, we examine DAA scale-up for both acute and chronic infection, as European [33] and US [34] guidelines recommend DAA therapy regardless of liver disease stage for HIV/HCV-coinfected individuals. However, if DAAs are prioritized or restricted to those with more advanced liver disease, then the prevention impact could be less than we predict. As such, the individual and population benefits achievable strongly support not restricting access to DAA therapy among HIV/HCV-coinfected MSM. Nevertheless, even if IFN-free DAA therapy is prioritized to those with advanced liver disease, it is possible that IFN-based treatment uptake among those with less advanced disease will remain high given historically high rates of uptake among HIV-coinfected MSM.

## CONCLUSIONS

We report a continuing HCV epidemic among HIV-diagnosed MSM in the United Kingdom, despite high rates of treatment, which is largely attributable to a high-risk population. Substantial reductions in HCV transmission within a decade could be achieved through rapid DAA scale-up and moderately successful behavioral interventions. This impact could be achieved despite reinfection rates that are roughly 5-fold higher than primary incidence, because the shortening and ease of delivery of new IFN-free DAAs enables scale-up with existing infrastructure. Given their importance in driving ongoing HCV transmission, there is a need to develop effective interventions to address high-risk behaviors associated with injecting and other drug use among MSM.

## Supplementary Data

Supplementary materials are available at http://cid.oxfordjournals.org. Consisting of data provided by the author to benefit the reader, the posted materials are not copyedited and are the sole responsibility of the author, so questions or comments should be addressed to the author.

Supplementary Data
