# Supplementary material for: Can Hepatitis C Virus (HCV) Direct-Acting Antiviral Treatment as Prevention Reverse the HCV Epidemic Among Men Who Have Sex With Men in the United Kingdom? Epidemiological and Modeling Insights
Source: Clin Infect Dis. 2016 Feb 16;62(9):1072–80. doi: 10.1093/cid/ciw075 (PMC4826456; doi:10.1093/cid/ciw075)
Supplement: Supplementary Data [file supp_62_9_1072__index.html]

Can Hepatitis C Virus (HCV) Direct-Acting Antiviral Treatment as Prevention Reverse the HCV Epidemic Among Men Who Have Sex With Men in the United Kingdom? Epidemiological and Modeling Insights — Can Hepatitis C Virus (HCV) Direct-Acting Antiviral Treatment as Prevention Reverse the HCV Epidemic Among Men Who Have Sex With Men in the United Kingdom? Epidemiological and Modeling Insights — Can Hepatitis C Virus (HCV) Direct-Acting Antiviral Treatment as Prevention Reverse the HCV Epidemic Among Men Who Have Sex With Men in the United Kingdom? Epidemiological and Modeling Insights — Supplementary Data 

# Can Hepatitis C Virus (HCV) Direct-Acting Antiviral Treatment as Prevention Reverse the HCV Epidemic Among Men Who Have Sex With Men in the United Kingdom? Epidemiological and Modeling Insights

## Supplementary Data

Supplementary Data

- Supplementary Data - Docx file
