# Supplementary material for: Can Hepatitis C Virus (HCV) Direct-Acting Antiviral Treatment as Prevention Reverse the HCV Epidemic Among Men Who Have Sex With Men in the United Kingdom? Epidemiological and Modeling Insights
Source: Clin Infect Dis. 2016 Feb 16;62(9):1072–80. doi: 10.1093/cid/ciw075 (PMC4826456; doi:10.1093/cid/ciw075)
Supplement: Supplementary Data [file supp_ciw075_ciw075supp.docx]

**SUPPLEMENTARY INFORMATION**

**Model fitting details:**

To incorporate parameter uncertainty, 1000 parameter sets were randomly sampled from the parameter distributions shown in **Table S1**. For each parameter set, the remaining parameters were varied to fit the model to observed epidemiological data using the methods of least squares from multiple start points. The parameters varied were:- proportion high-risk, relative risk of high-risk compared to low-risk, infection rate, HCV prevalence among the low- and high-risk groups in 1996, proportion HCV diagnosed in the acute HCV stage, and HCV treatment rate after the first year of diagnosis.

**Sensitivity analyses:**

We explore the impact of: 85% or 95% SVR with future treatments (90% at base-case), DAA retreatment eligibility after reinfection (no retreatment or retreatment with IFN/RBV compared to retreatment with DAAs at base-case), a linear increase in testing from 2015 until 100% are tested annually (stable rates of testing after 2015 at 88% base-case), reductions in risk post-treatment of 50%/100% from 2015 (no reductions in base-case), partial assortative mixing between high/low-risk groups (50% assortative, proportional in base-case), and seeding of HCV from outside the diagnosed HIV-positive population such as from HIV-negative MSM (constant incidence of HCV infections of 1.5 per 1000 pyrs, similar to HCV incidence amongst HIV-negative MSM ([1](#_ENREF_1), [2](#_ENREF_2))) which is unaffected by treatment. Additionally we examine the additional benefit of a short-term (<1 year) behavior change after HCV diagnosis. For this analysis, we model a 20% reduction in risk behavior after diagnosis for up to 1 year (for those who are not treated within the first year) or until HCV treatment.

**Supplementary Table S1. Model parameterization**

| **Sampled Parameter** | **Mean Value [sampled range, distribution]** | **Source** |
| --- | --- | --- |
| **Entry rate of newly HIV-diagnosed MSM** | Varied over calendar time (varies between 1500 and 2900 per year) | Estimated through linear curve fit to annual reports of new HIV diagnoses in the UK from 1999-2007, with constant rates post 2007 and from 1996-1999 based on UK data (PHE HIV in the UK reports). |
| **Life expectancy from HIV diagnosis** | Varies over calendar time based on increasing ART coverage and earlier diagnosis (20-40 years from assumed HIV diagnosis and ART initiation at age 35([3](#_ENREF_3))) | Average age at HIV diagnosis in the UK is 34 years([3](#_ENREF_3)). We fit a linear curve to life-expectancy data among diagnosed individuals who start ART at age 35 from 1997-2008 over calendar time among UK cohort([4](#_ENREF_4)). Uncertainty was included in these fits by sampling each life-expectancy point estimate from the distribution reported in the study and refitting the linear curves. We assume a continued increase in life expectancy from 2008 until a life expectancy of 75 years is reached (consistent with model estimates of life expectancy in a high diagnosis setting ([5](#_ENREF_5)), and achieved in 2010 in the model) |
| **Excess liver-related mortality due for those with chronic HCV (annual)** | 0.16 per 100 person-years [uniform 0.05-0.27] | ([6](#_ENREF_6), [7](#_ENREF_7)) |
| **Proportion of HIV-positive who spontaneously clear acute HCV infection** | 15% [10-20%, uniform] | ([8](#_ENREF_8), [9](#_ENREF_9)) and UK CHIC data |
| **Duration acute infection until spontaneous clearance** | 6 months [3-9 months, uniform] | ([9](#_ENREF_9)) |
| **Proportion of those undiagnosed who are HCV tested (Ab or RNA) each year** | Varies over calendar time up to 88% per year in 2015 | Calculated from linear fit to UK CHIC data in supplementary table 1, assuming a linear increase in testing from 0% in 1996. After 2015, we assume no increase in testing in the base-case scenario, and continued increase to 100% per year in the sensitivity analysis. |
| **Proportion of those infected who do not spontaneously clear initiated onto treatment within 1 year of diagnosis** | After acute HCV diagnosis (excluding those who spontaneously clear the virus): 46% (104/224)(40-53)  After chronic diagnosis: 22% (247/1103) (20-24) | Calculated from UK CHIC data, with the denominator for the acute stage adjusted to exclude those who proceed to spontaneous clearance and therefor are not ‘eligible’ for treatment in the model. Assume a fixed rate of treatment from 2003 onwards, as the proportion of eligible individuals treated in UK CHIC (465/997) remained stable for those diagnosed between 2004-2007 (44.3% eligible treated [95%CI 40-49%]) compared to those diagnosed between 2008-2011 (48.6% [95%CI 44-53]). Prior to 2003, few were treated and SVR rates were likely very low in this period. |
| **SVR with IFN/RBV**  **<1 year from HCV infection**  **(‘Acute HCV’)**  **>1 year from HCV infection**  **(‘Chronic HCV’)** | 80% [70-90%, uniform]  30% [25-35%, uniform] | ([9-12](#_ENREF_9))  Weighted based on genotype distribution in UK CHIC (89% genotype 1 and 11% genotype 2/3 from UK CHIC) and SVR by genotype from a recent meta-analysis([13](#_ENREF_13))). |
| **SVR with DAAs** | 90% | Assumed |
| **Year of HCV epidemic seeding** | 1996 | ([14](#_ENREF_14)) |
| **Proportion of individuals infected with HCV upon HIV diagnosis** | 0.5% [0-1%, uniform] | Assumed based on the prevalence of HCV among HIV-negative MSM in the UK ([15](#_ENREF_15)) and a European estimate of the prevalence of HV among HIV-positive undiagnosed MSM([16](#_ENREF_16))) |
| **Parameters used for model fitting (NOT SAMPLED, point estimate used)** | | **Source** |
| **HCV incidence among diagnosed HIV-positive MSM (by year)** | Table 2 | UK CHIC |
| **HCV prevalence (Ab+ or RNA+) among diagnosed HIV-positive MSM (by year)** | Table 1 | UK CHIC |
| **HCV reinfection incidence after treatment or spontaneous clearance** | 2004-2012: 7.8 per 100 py (95%CI 5.8-10.5) | ([11](#_ENREF_11)) |
| **Number of diagnosed MSM in 2013** | 36,310 (95% CI 35,610-37,090) | ([3](#_ENREF_3)) |
| **Cumulative proportion HCV diagnosed ever treated by 2011** | 44% (586/1327) | Calculated from UK CHIC data, with the denominator adjusted to exclude those who proceed to spontaneous clearance and therefore are not ‘eligible’ for treatment in the model. |
| **Parameters which were varied for the fitting** | **Prior range/ Posterior mean [95% Interval]** | **Comments** |
| **Proportion high risk** | Prior range: 0-25%  Posterior: 7% [95%I 3-14%] | In 2010 European MSM Internet Survey [EMIS], 8% of HIV-positive MSM in the UK report methamphetamine use in the previous 4 weeks (unpublished). Among a cross-sectional study of HIV+ MSM in the UK, 8% reported injecting drug use in the past 5 years, and 13% reported fisting([17](#_ENREF_17)). |
| **Relative risk high risk compared to low risk** | Prior range: 0-100  Posterior: 48 [95%I 22-90] | Fitted value higher than reported risk of sex with methamphetamine (Adjusted odds ratio 28.6 [95%CI 1.84-443]([18](#_ENREF_18))) and reporting at least three sex risk factors [receptive unprotected anal intercourse[UAI], insertive UAI, receptive fisting, or insertive fisting] (Adjusted odds ratio 23.5 (95%CI 9.5-58.3)([19](#_ENREF_19))) though within the uncertainty bounds. |
| **Leaving rate from high risk (annual)** | Prior range: 0-1  Posterior: 0.12 [95%I 0-0.26] |  |
| **Initial HCV prevalence in 1996**  **Low risk**    **High risk** | Prior range: 0-2%  Posterior: 1.2% [95%I 0.1-2%]  Prior range: 0-50%  Posterior: 31% [95%I 22-49%] |  |
| **Infection rate** | Prior range: 0-1  Posterior: 0.02 [95%I 0.005-0.028] |  |
| **Treatment rate >1 year post-diagnosis (annual)** | Prior range: 0-20%  Posterior: 6.8% [95%I 3.8-9.9] | Assume a fixed rate of treatment from 2003 onwards, as the proportion of eligible individuals treated in UK CHIC (465/997) remained stable for those diagnosed between 2004-2007 (44.3% eligible treated [95%CI 40-49%]) compared to those diagnosed between 2008-2011 (48.6% [95%CI 44-53]). Prior to 2003, few were treated and SVR rates were likely very low in this period. |
| **Number of diagnosed MSM in 1996** | Prior range: 0-10,000  Posterior: 6735 [6300-7381] |  |

**Supplementary Table S2**: Proportion of individuals who are not previously known to be HCV infected who are tested for HCV antibody within a given year among the UK CHIC cohort

| Year | Number of MSM in follow-up in that year who are not already known to be HCV positive | Number tested within year for HCV antibody | Proportion tested for HCV-antibody |
| --- | --- | --- | --- |
| 2004 | 10610 | 3261 | 30.74% |
| 2005 | 11257 | 4290 | 38.11% |
| 2006 | 11639 | 4666 | 40.09% |
| 2007 | 12088 | 5828 | 48.21% |
| 2008 | 12324 | 6902 | 56.00% |
| 2009 | 12601 | 7633 | 60.57% |
| 2010 | 12825 | 7922 | 61.77% |
| 2011 | 11904 | 7786 | 65.41% |

### Supplementary Table S3. Number of individuals with any evidence of treatment

| HCV infection | Total | Treated | % |
| --- | --- | --- | --- |
| Total | 1367 | 586 | 42.8% |
| Acute HCV infection at diagnosis | 264 | 133 | 50.4% |
| No evidence of acute infection at diagnosis | 1103 | 453 | 41.1% |

### Supplementary Table S4. Time between diagnosis and first treatment

| Time from first positive to first treatment | Total number of individuals | % | Acute infection at diagnosis | % | No evidence of acute infection at diagnosis | % |
| --- | --- | --- | --- | --- | --- | --- |
| Year <1 | 351 | 59.9% | 104 | 78.2% | 247 | 54.5% |
| 1<Year<2 | 58 | 9.9% | 18 | 13.5% | 40 | 8.8% |
| 2<Year<3 | 41 | 7.0% | 4 | 3.0% | 37 | 8.2% |
| 3<Year<4 | 27 | 4.6% | 4 | 3.0% | 23 | 5.1% |
| 4<Year<5 | 24 | 4.1% | 1 | 0.8% | 23 | 5.1% |
| >5 years | 85 | 14.5% | 2 | 1.5% | 83 | 18.3% |

**Supplementary Figure S1. One-way sensitivity analyses showing model projections of mean chronic HCV prevalence among HIV-infected MSM in 2025 with scale-up of HCV treatment (80% <1 year from diagnosis, 20% thereafter) with DAAs from 2015.**


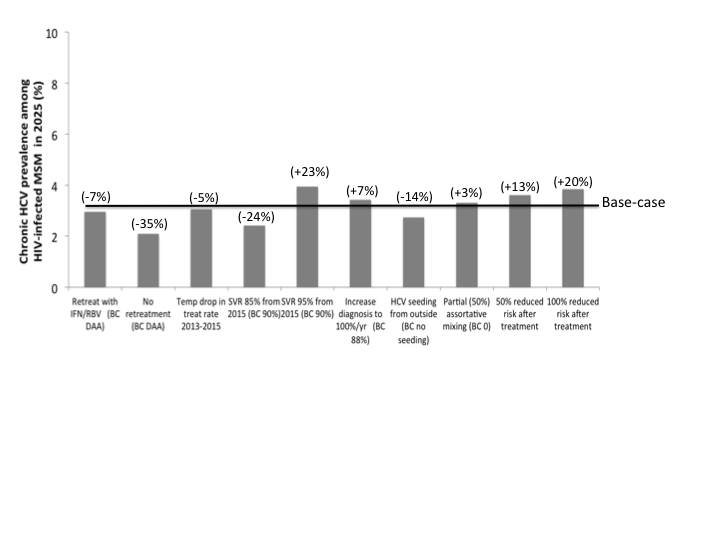


1. Yaphe S, Bozinoff N, Kyle R, Shivkumar S, Pai N, Klein M. Incidence of acute hepatitis C virus infection among men who have sex with men with and without HIV infection: a systematic review. Sex Trans Infec. 2012;88(7):558-64.

2. Richardson D, Fisher M, Sabin CA. Sexual Transmission of Hepatitis C in MSM May Not Be Confined to Those with HIV Infection. Journal of Infectious Diseases. 2008 April 15, 2008;197(8):1213-4.

3. Public Health England. HIV in the United Kingdom: 2014 Report. 2014.

4. May M, Gompels M, Delpech V, Porter K, Post F, Johnson M, et al. Impact of late diagnosis and treatment on life expectancy in people with HIV-1: UK Collaborative HIV Cohort (UK CHIC) Study2011 2011-10-11 22:34:39.

5. Nakagawa F, Lodwick R, Smith C, Ssmith R, Cambiano V, Lundgren JD, et al. Projected life expectancy of people with HIV according to timing of diagnosis. AIDS. 2012;26(3):335-43.

6. van der Helm J, Geskus R, Sabin C, Meyer L, del Amo J, Chêne G, et al. Effect of HCV Infection on Cause-Specific Mortality After HIV Seroconversion, Before and After 1997. Gastroenterology. 2013 4//;144(4):751-60.e2.

7. Weber R, Sabin C, Friis-Møller N, Reiss P, El-Sadr W, Kirk O, et al. Liver-related deaths in persons infected with the human immunodeficiency virus: the D:A:D study. Arch Intern Med. 2006;166(15):1632-41.

8. Thomson EC, Fleming VM, Main J, Klenerman P, Weber J, Eliahoo J, et al. Predicting spontaneous clearance of acute hepatitis C virus in a large cohort of HIV-1-infected men. Gut. 2011 June 1, 2011;60(6):837-45.

9. Piroth L, Larsen C, Binquet C, Alric L, Auperin I, Chaix M-L, et al. Treatment of acute hepatitis C in human immunodeficiency virus–infected patients: The HEPAIG study. Hepatology. 2010;52(6):1915-21.

10. Webster DP, Wojcikiewicz T, Keller M, Castelnovo D, Mistry H, Gilleece Y, et al. Spontaneous clearance and treatment of acute hepatitis C infection in HIV-positive men with 48 weeks of interferon-alpha and ribavirin. International Journal of STD & AIDS. 2013 March 1, 2013;24(3):179-83.

11. Martin T, Martin N, Hickman M, Vickerman P, Page E, Everett R, et al. HCV reinfection incidence and treatment outcome among HIV-positive MSM in London. AIDS. 2013;doi:10.1097/QAD.0b013e32836381cc

12. Wandeler G, Schlauri M, Jaquier M-E, Rohrbach J, Metzner KJ, Fehr J, et al. Incident Hepatitis C Virus Infections in the Swiss HIV Cohort Study: Changes in Treatment Uptake and Outcomes between 1991 and 2013. Open Forum Infectious Diseases. 2015 February 19, 2015.

13. Davies A, Singh KP, Shubber Z, duCros P, Mills EJ, Cooke G, et al. Treatment Outcomes of Treatment-Naïve Hepatitis C Patients Co-Infected with HIV: A Systematic Review and Meta-Analysis of Observational Cohorts. PLoS ONE. 2013;8(2):e55373.

14. van de Laar T, Pybus O, Bruisten S, Brown D, Nelson M, Bhagani S, et al. Evidence of a large, international network of HCV transmission in HIV-positive men who have sex with men. Gastroenterology. 2009 May;136(5):1609-17. PubMed PMID: 19422083. Epub 2009/05/08. eng.

15. Turner J, Gikanga T, Jones V, Copas A, Cooper J, Stevens J, et al. Is there an unrecognized epidemic of HCV among men who have sex with men? . 14th Annual Conference of the British HIV Association (BHIVA); 23-25 April 2008, Belfast Waterfront Hall, Northern Ireland2008.

16. Schmidt A, Falcato L, Zahno B, Burri A, Regenass S, Mullhaupt B, et al. Prevalence of hepatitis C in a Swiss sample of men who have sex with men: whom to screen for HCV infection. BMC Public Health. 2014;14(3):doi:10.1186/471-2458-14-3.

17. Turner JM, Rider AT, Imrie J, Copas AJ, Edwards SG, Dodds JP, et al. Behavioural predictors of subsequent hepatitis C diagnosis in a UK clinic sample of HIV positive men who have sex with men. Sexually Transmitted Infections. 2006 August 1, 2006;82(4):298-300.

18. Fierer D, Factor S, Uriel A, Mullen M, Klepper A, van Seggelen W, et al. Sexual Transmission of Hepatitis C Virus Among HIV-infected Men Who Have Sex with Men - New York City 2005-2010. Morbidity and Mortaility Weekly Report. 2011;60(28):945-50.

19. Danta M, Brown D, Bhagani S, Pybus OG, Sabin C, Nelson M, et al. Recent epidemic of acute hepatitis C virus in HIV-positive men who have sex with men linked to high-risk sexual behaviours. AIDS. 2007;21:983-91.
